# Nanostructured Semiconductors for Flexible Thermoelectric Applications

**DOI:** 10.3390/nano15241843

**Published:** 2025-12-08

**Authors:** Yi Luo, Chengxuan Yu, Yuanbin Niu, Haoyi Guo, Xiaobin Feng

**Affiliations:** Hubei Key Laboratory of Theory and Application of Advanced Materials Mechanics, School of Physics and Mechanics, Wuhan University of Technology, Wuhan 430070, China

**Keywords:** nanostructured semiconductors, low-dimensional, defect engineering, strength-ductility synergy, flexible thermoelectric applications

## Abstract

The development of miniaturized, integrated, and flexible thermoelectric devices has intensified the demand for high-performance thermoelectric semiconductors. While significant advances have been made in optimizing their thermoelectric properties, mechanical performance in terms of the strength and ductility has remained a challenge. Consequently, the inherent brittleness and insufficient mechanical robustness of inorganic thermoelectric semiconductors present a major barrier to their commercial applications. Therefore, it is essential to develop thermoelectric materials with enhanced reliability and operational lifespan of flexible thermoelectric devices. This review summarizes recent breakthroughs in low-dimensional thermoelectric materials and emerging defect engineering strategies, which offer promising pathways for simultaneously improving both mechanical and thermoelectrical performance. By precisely regulating the relationship between nanostructural design and performance characteristics, new opportunities are emerging for nanostructured semiconductors in flexible thermoelectric applications across wide temperature ranges, from near-ambient to elevated conditions.

## 1. Introduction

The sustainable utilization and efficient conversion of energy stand as one of the global challenges in the 21st century [1]. Thermoelectric (TE) semiconductors, which enable direct conversion between thermal and electrical energy through the Seebeck and Peltier effects, have attracted considerable interest due to their potential in energy harvesting and solid-state refrigeration [2]. These semiconductors demonstrate significant application potential in diverse fields, including space power systems, industrial waste heat recovery, and localized precision thermal management [3]. Nevertheless, despite their remarkable TE properties, the generally poor mechanical properties of most TE materials severely limit their widespread commercial applications [4]. Therefore, developing TE materials and devices that integrate outstanding mechanical robustness with thermoelectric performance has become a critical research frontier for advancing TE conversion technologies [5,6].

The main challenge in advanced TE semiconductors lies in achieving the concurrent optimization of their thermoelectrical and mechanical properties [7,8]. The energy conversion efficiency of a TE material is governed by its dimensionless figure of merit, *ZT* (*ZT* = *S^2^σT*/*κ*, where *S* denotes the Seebeck coefficient, *σ* is the electrical conductivity, *κ* is the thermal conductivity, and *T* is the absolute temperature) [9,10]. This relationship highlights the ideal characteristics of a high-performance TE semiconductor: a large Seebeck coefficient, high electrical conductivity, and low thermal conductivity. However, the strong interdependence among these parameters complicates the optimization process [9,10]. Furthermore, for flexible applications, TE materials must maintain both structural integrity and functional stability under repeated mechanical deformation. Specifically, on one hand, insufficient strength to withstand external stress directly compromises manufacturing processes such as material shaping, micromachining, and device integration [8]. On the other hand, adequate ductility is essential to accommodate various deformation modes encountered in flexible devices, including tension, compression, and cyclic bending or twisting [11]. Therefore, the strength-ductility synergy is critical to ensuring the reliability and longevity of flexible TE systems in practical applications. In response to these challenges, nanostructured TE semiconductors have emerged as a promising direction in recent studies.

These innovative architectures can be categorized into two distinct types: external nanostructures, exemplified by low-dimensional TE semiconductors, and internal nanostructures, realized through various defect engineering strategies. In low-dimensional TE semiconductors, the reduction in characteristic dimensions to the micro/nanoscale leads to a substantial decrease in bending strain, thereby conferring intrinsic flexibility [11,12]. Apart from the inherent mechanical advantages of dimensional scaling, as realized in nanowires, thin films, and pillars, quantum confinement effects contribute to enhanced power factor (*S*^2^*σ*). Concurrently, their abundant surfaces and interfaces promote phonon scattering, thereby reducing the lattice thermal conductivity *κ_L_* [13]. A representative example is Bi_2_Te_3_ films fabricated on flexible substrates, which exhibit both high thermoelectric performance and remarkable bending stability due to their optimized two-dimensional nanostructure [3,9].

Defect engineering has emerged as another pivotal strategy for optimizing thermoelectric semiconductors through precisely controlled micro/nanostructures, primarily implemented via three defect categories: dislocations, boundaries, and nanocomposites. Dislocations serve as effective phonon scattering centers to reduce *κ_L_*, while simultaneously enhancing mechanical properties through mechanisms such as pinning, multiplication, and motion that suppress crack propagation and improve strength and ductility, as demonstrated in SnTe with high-density dislocation networks [14]. Boundary Engineering, such as grain boundaries, stacking faults, and twins, not only scatters phonons but also impedes dislocation motion or acts as the dislocation source to accommodate strain [15], exemplified by the significantly increased shear strength in Bi_2_Te_3_ by high-density nanotwins [16] and stacking faults [17]. Anti-site defects and kink structures can also generate and store high-density dislocations and effectively hinder crack propagation, improving fracture toughness [18,19]. Nanocomposites, including nanoprecipitates (e.g., MnTe precipitates in SnTe) [20,21], heterostructures (e.g., *α*/*β* dual-phase Cu_2_Se) [22], and porous architectures (e.g., porous Bi_2_Te_3_ and SnTe-based semiconductors) [23,24,25], enhance strength and toughness via crack bridging, deflection, and pinning, while simultaneously reducing thermal conductivity through intensified phonon scattering at interfaces.

From an application perspective, flexible thermoelectric materials can be categorized into near-room-temperature and high-temperature types based on their operating ranges. Typically, TE semiconductors configured as nanowires, nanotubes, and thin films are used in near-room-temperature applications. For example, vertical silicon nanowire arrays with thermal conductivity approaching the amorphous limit have been developed, enabling the realization of macroscopic silicon-based thermoelectric devices with relatively high output power density and achieving a power factor exceeding 5 mW·m^−1^·K^−2^ at room temperature [26]. Additionally, high-performance PEDOT/Ag_2_Se/CuAgSe flexible composite films have been fabricated, allowing the construction of thermoelectric generators that effectively harness the human body’s temperature difference and providing innovative solutions for wearable electronics [27]. In contrast, high-temperature flexible thermoelectric materials often exist in bulk forms with generally larger dimensions and find broader application in industrial settings. A prominent example is Ag_2_S-based semiconductors engineered into high-performance flexible thermoelectric systems. This approach has yielded all-inorganic flexible thermoelectric generators demonstrating a remarkable normalized maximum power density of 0.08 W·m^−1^ [28].

**Figure 1:**
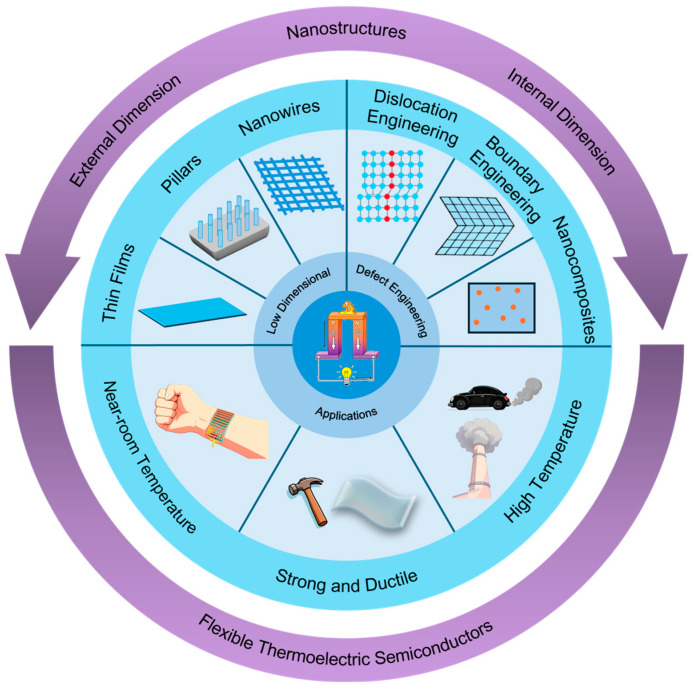
**A schematic diagram of nanostructured semiconductors for flexible thermoelectric applications.** Novel nanostructures, particularly low-dimensional thermoelectric semiconductors and defect engineering, offer promising pathways for concurrently enhancing both mechanical and thermoelectric performance, endowing new opportunities for nanostructured semiconductors in flexible thermoelectric applications across a wide temperature range, from near-room temperature to high temperature.

This review begins with a systematic overview of novel nanostructures, such as low-dimensional thermoelectric semiconductors and defect engineering, delving into how external dimensional design and internal nanostructural modulation synergistically optimize mechanical and thermoelectric performance. Subsequently, the review highlights application breakthroughs in flexible thermoelectrics across a broad temperature range, from near-room temperature to high temperature, as shown in Figure 1. Ultimately, this review aims to not only catalog key advances in nanostructural design and multi-scenario applications but also to establish a foundation for future innovation in high-performance flexible thermoelectric technology.

## 2. Low-Dimensional TE Semiconductors

The physical and chemical properties of materials become strongly dependent on geometric size as their characteristic dimensions shrink to the micro- or nanoscale, denoted as the size effect [29]. This includes external size effects, where properties are influenced by external dimensions such as film thickness, nanowire diameter, and pillar diameter [30]. A fundamental transition occurs when these external dimensions approach or fall below the characteristic length scales of internal mechanisms, such as the mean free paths of electrons and phonons or the critical length for dislocation activation, leading to profound changes in material behaviors [31,32]. For TE semiconductors, this principle provides a powerful strategy for the concurrent optimization of both mechanical robustness and thermoelectric efficiency.

### 2.1. Nanowires

**Figure 2:**
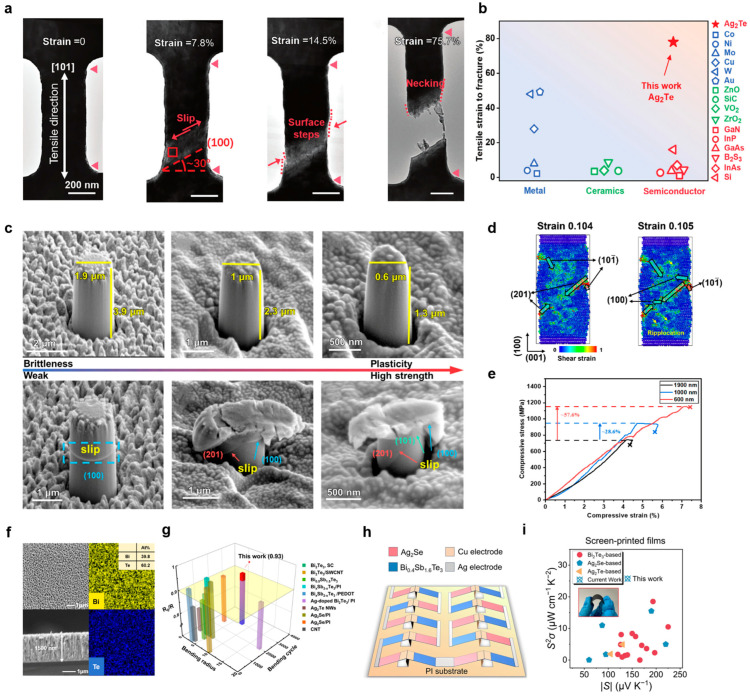
**Low-dimensional TE semiconductors, including nanowires, pillars, and thin films.** (**a**) Typical microstructural evolution and corresponding mechanisms of Ag_2_Te nanowires at different strain stages: undeformed (0%), slip-dominated deformation (7.8%), surface step formation (14.5%), necking and fracture (75.7%). The two red triangles in the image serve as strain gauges for the strain measurement [6]. (**b**) A comparison of tensile fracture strain reveals that single-crystal Ag_2_Te nanowires under uniaxial tension outperform other micro/nano-scale metals, ceramics, and semiconductors [6]. (Reprinted with permission from ref. [6]. Copyright 2025 Wiley-VCH Verlag GmbH & Co. KGaA, Weinheim, Germany). (**c**) Size effects on mechanical failure by comparing the microstructural evolution of single-crystalline SnSe micropillars (diameters: 600, 1000, and 1900 nm) before and after compression [38]. (**d**) MD simulations of the structural deformation in a 10 nm SnSe pillar under compressive loading. Atomic configurations at strains of 0.104 and 0.105 [38]. (**e**) Compressive stress–strain curves of SnSe pillars with varying diameters [38]. (Reprinted with permission from ref. [38]. Copyright 2025 Elsevier Ltd.). (**f**) SEM analysis of a Bi_2_Te_3_ film surface and cross-section [47]. (**g**) Conductivity vs. bending ratio/cycles in stacking-faulted Bi_2_Te_3_ films: a comparison with other flexible thermoelectric films [47] (Reprinted with permission from ref. [47]. Copyright 2025 Wiley-VCH Verlag GmbH & Co. KGaA, Weinheim, Germany). (**h**) A schematic diagram of the Ag_2_Se film thermoelectric device structure [49]. (**i**) A comparison of the power factor versus Seebeck coefficient between the Ag_2_Se film and previously reported near-room-temperature inorganic films fabricated via screen-printing. The inset illustrates the bending flexibility of the Ag_2_Se film [49] (Reprinted with permission from ref. [49]. Copyright 2025 The Author(s). Published by Springer Nature under a Creative Commons Attribution 4.0 International License).

Low-dimensional nanowires enable the synergistic optimization of electrical, thermal, and mechanical properties in thermoelectrics through the size effect. In Ag_2_Te nanowires, for instance, diameter reduction to the nanoscale leads to quantum confinement, which modifies the electronic band structure to enhance the Seebeck coefficient without compromising electrical conductivity [33]. In situ transmission electron microscopy (TEM) mechanical testing on Ag_2_Te nanowires further reveals a transition in deformation mechanisms at the nanoscale, where processes like dislocation slip, deformation twinning, and Ag nanobridge formation facilitate an exceptional tensile fracture strain of 75.7% (Figure 2a). As quantitatively compared in Figure 2b, this ductility markedly exceeds that of most other micro- and nanoscale materials [6]. In III-V semiconductors such as GaAs, in situ mechanical testing has vividly captured this brittle-to-ductile transition at the nanoscale, which stems from a reduced density of crack initiation sites coupled with surface-stress-dominated dislocation activity [32]. Low-dimensional Si, SiGe, and InAs nanowires provide an effective pathway for optimizing thermoelectric transport properties through inherent size effects and tunable surface states. Dimensional confinement and interface scattering mechanisms, such as surface roughness in Si nanowires and alloy scattering in SiGe nanowires, selectively suppress thermal conductivity while largely preserving electrical performance [34,35,36,37]. For instance, rough silicon nanowires can dramatically reduce their lattice thermal conductivity close to the amorphous limit while maintaining the power factor, leading to a notable enhancement in *ZT* [34]. Collectively, this nanowire-based low-dimensional design and structural engineering establish a material foundation for high-performance thermoelectric devices.

### 2.2. Pillars

Although both nanopillars and nanowires are one-dimensional nanostructures with high aspect ratios, they differ fundamentally in their fabrication and structural characteristics. Nanopillars are typically defined as pillar-shaped structures with regular morphology and ordered arrays, fabricated via top-down microprocessing techniques such as etching and electrodeposition. In contrast, nanowires are generally synthesized through bottom-up methods like vapor-liquid-solid (VLS) growth.

Low-dimensional pillars represent a unique pathway for optimizing mechanical properties via external size effects. Recent studies on SnSe pillars demonstrated that reducing their external dimensions can simultaneously enhance both strength and ductility [38]. A comparative analysis of scanning electron microscopy (SEM) images before and after compression (Figure 2c), combined with molecular dynamics simulations (Figure 2d), reveals that below a critical size, the activation of multiple slip systems suppresses brittle fracture while promoting dislocation-mediated plastic flow. Consistent with this mechanism, the stress–strain curves in Figure 2e quantitatively show that as the diameter of SnSe pillars decreases, their strength increases from 0.73 to 1.15 GPa and plasticity from 4.4 to 7.3%. A similar mechanical transition has also been observed in silicon nanopillars below critical diameters [31].

Besides excellent mechanical properties, nanopillars also offer significant advantages in integration density and manufacturing consistency. For instance, using pulsed electroplating technology to deposit Bi_2_Te_3_ and Sb_2_Te_3_ into the through-holes of a glass template, high-aspect-ratio thermoelectric micropillars can be fabricated. This approach has enabled the integration of four thermocouples within a single device, achieving a record temperature difference of 138 K and a high output voltage of 10.22 mV per thermocouple. These results fully demonstrate the reproducibility and scalability of the top-down approach for realizing high-density, high-performance thermoelectric systems [39]. Moreover, Ag_2_Se micropillars synthesized via solvothermal methods exhibit highly localized lattice distortions and strain fields. Theoretical predictions suggest that their *ZT* value could reach 1.4 after carrier concentration optimization, highlighting the great potential of micropillar structures in next-generation efficient micro-thermoelectric devices [40].

### 2.3. Thin Films

In TE thin film systems, precise control over external dimensions can induce significant microstructural and property modifications [41,42,43,44,45]. In recent years, quasi-layered and two-dimensional (2D) van der Waals thermoelectric bulk semiconductors have demonstrated remarkable ductility. A notable example is *α*-Ag_2_S, which exhibits a compressive engineering strain exceeding 50% and a bending engineering strain above 20% [46]. The weak van der Waals interactions between layers in such materials not only enable exceptional mechanical deformability but also suppress cross-plane thermal conductivity. Together with band convergence and enhanced interface phonon scattering, these factors collectively contribute to an improved *ZT* [3]. Mechanical exfoliation of 2D thermoelectric bulk materials has opened new avenues for high-performance flexible thin films. A representative example is the “staggered-layer” structure obtained through mechanical exfoliation of Bi_2_Te_3_ single crystals, which simultaneously enhances mechanical flexibility and electrical performance, maintaining 91% of its original electrical conductivity after 1000 bending cycles while achieving a room-temperature power factor of 4.2–4.6 mW m^−1^ K^−2^ [9].

Meanwhile, the film deposition is another approach for achieving high-performance thermoelectric thin films. Magnetron-co-sputtered Bi_2_Te_3_ films with coordinated grain size and stacking fault control achieve both mechanical synergy (~363 MPa yield strength with ~7.3% tensile strain) and high power factor (2760 μW m^−1^ K^−2^) through boundary engineering (Figure 2f) [47]. These films also maintain exceptional electrical conductivity stability during repeated bending (Figure 2g), underscoring their suitability for flexible thermoelectric applications [47]. Annealing promoted grain growth from 18.5 to 34.2 nm in Bi_2_Te_3_ films, and this microstructural evolution significantly increased both carrier mobility and concentration, thereby reducing electrical resistivity [48]. Through multi-scale structural design, the Ag_2_Se film exhibits enhanced power factor and Seebeck coefficient compared to screen-printed counterparts (Figure 2h), along with confirmed mechanical flexibility. Device integration using a triangular p-n junction architecture (Figure 2i) enabled increased leg length within a compact space, thereby boosting power density and cooling performance [49]. These findings demonstrate that the control of intrinsic interfaces and extrinsic defects in thin films can effectively achieve mechanical robustness and high thermoelectrical performance, as shown in Table 1.

## 3. Defect Engineering

### 3.1. Dislocation Engineering

**Figure 3:**
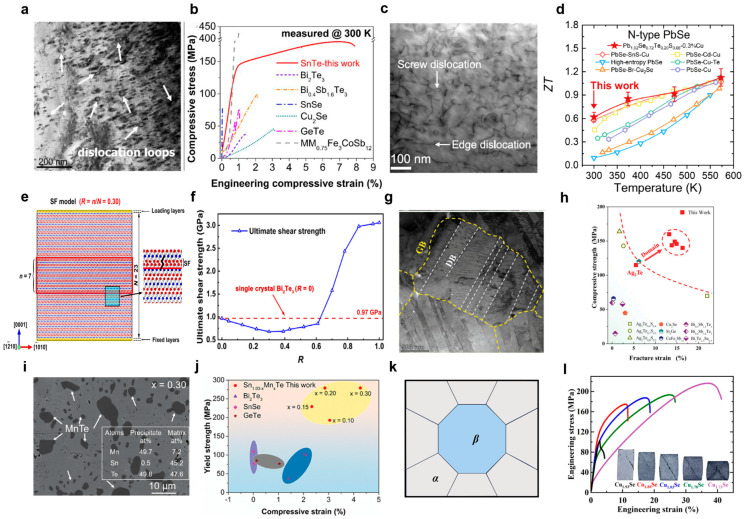
**Defect Engineering, including dislocation engineering, boundary engineering, and Nanocomposites.** (**a**) A representative low-magnification TEM image of SnTe, all arrows indicate dislocation loops [14]. (**b**) Room-temperature compressive performances: SnTe versus conventional brittle bulk TE materials [14] (Reprinted with permission from ref. [14]. Copyright 2023 Elsevier Ltd.). (**c**) The scanning TEM image of n-type PbSe [53]. (**d**) Plot of *ZT* values as a function of temperature [53] (Reprinted with permission from ref. [53]. Copyright 2022 The Author(s). Published by Springer Nature under a Creative Commons Attribution 4.0 International License). (**e**) The stacking fault model with a density of 0.30 [17]. (**f**) Ultimate shear strength of models with different stacking fault densities. The red dashed line represents the strength of single-crystal Bi_2_Te_3_ [17] (Reprinted with permission from ref. [17]. Copyright 2023 Elsevier Ltd.). (**g**) The domain structure of the Ag_2_Te-50% hot deformed sample [60]. (**h**) The compressive strength vs. fracture strain of the Ag_2_Te at room temperature [60] (Reprinted with permission from ref. [60]. Copyright 2023 Wiley-VCH Verlag GmbH & Co. KGaA, Weinheim, Germany). (**i**) SEM image of the x = 0.30 sample; inset shows the EDS analysis of the matrix and precipitates [20]. (**j**) Room-temperature compressive performance of the Sn_0.93_Mn_0.10_Te sample compared with that of several state-of-the-art thermoelectric materials [20] (Reprinted with permission from ref. [20]. Copyright 2024 Wiley-VCH Verlag GmbH & Co. KGaA, Weinheim, Germany). (**k**) Schematic of heterostructured Cu_2−x_Se before compressive deformation [22]. (**l**) Engineering stress–strain curves of Cu_2−x_Se (x = 0.07, 0.12, 0.17, 0.22, 0.27). The inset shows optical images of the Cu_2−x_Se sample after the compression test [22] (Reprinted with permission from ref. [22]. Copyright 2025 Elsevier Ltd.).

Dislocation engineering has emerged as a pivotal strategy for unlocking the intrinsic plasticity of semiconductors. However, in most inorganic thermoelectric materials, strong covalent/ionic bonding, complex crystal structures, and limited slip systems create high energy barriers for dislocation nucleation and motion, leading to inherent brittleness [50]. Dislocation engineering addresses this challenge by deliberately introducing and activating mobile dislocations, thereby unlocking intrinsic plasticity. A key advantage of this strategy over those relying on external size effects is its direct applicability to bulk materials, underscoring its broad relevance for mechanical enhancement. For example, SnTe, a relatively more environmentally friendly thermoelectric semiconductor than its lead-based analogues (e.g., PbTe), has attracted considerable attention [51]. Through a process involving melt quenching and spark plasma sintering, high-density dislocation networks can be introduced into SnTe bulk samples. These networks effectively accommodate plastic strain and suppress crack propagation, resulting in a compressive strain of ~7.5% and a yield strength of ~180 MPa, as shown in Figure 3b [14].

In addition to mechanical robustness, dislocation engineering also offers a promising route to enhancing thermoelectric properties. For instance, the high-density dislocations introduced in BiCuSeO oxide ceramics via ultra-high pressure sintering effectively scatter phonons, reducing the lattice thermal conductivity to 0.13 W·m^−1^·K^−1^ at 767 K and yielding a *ZT* value of 1.69 [52]. Similarly, in n-type PbSe-based materials, doping-induced dense dislocations contribute to a high *ZT* of 0.96 in the low-to-medium temperature range [53]. The corresponding scanning transmission electron microscopy (STEM) image (Figure 3c) from n-type PbSe work provides direct evidence of dislocation structures. Meanwhile, Figure 3d plots the corresponding high *ZT* values as a function of temperature. Furthermore, the Seebeck coefficient becomes three times higher than that of the as-prepared Bi-Te nanowires, which is attributed to an increased concentration of crystal defects, particularly edge dislocations [54]. These examples highlight the potential of dislocation engineering to enhance both mechanical and thermoelectric properties.

### 3.2. Boundary Engineering

Boundary engineering involves the design of two-dimensional defects where atomic stacking sequences or crystal orientations change, including grain boundaries [47], stacking faults [55,56], twin boundaries [15,57,58,59], domain boundaries [60], and phase boundaries [61]. From a mechanical perspective, these boundaries act as barriers that impede dislocation motion to provide strengthening, while specific boundaries (e.g., coherent twin boundaries) can also nucleate dislocations to accommodate strain, thereby enhancing ductility and avoiding brittle fracture [62,63]. In terms of thermoelectric performance, the interfaces effectively scatter phonons, particularly in the mid- to high-frequency range, leading to a substantial suppression of lattice thermal conductivity [55,64].

High-density stacking faults have been demonstrated to markedly enhance shear strength by inducing crystal structure reconstruction. For instance, molecular dynamics simulations demonstrate that in Bi_2_Te_3_ a high density of stacking faults (>70%) triggers structural reorganization, forming a complete fault network that strengthens interlayer interactions (Figure 3e), resulting in a quantitatively demonstrated boost in strength, as depicted in Figure 3f [17]. Similarly, the introduction of high-density nanoscale domain boundaries into Ag_2_Te through hot deformation leads to concurrent mechanical and thermoelectric enhancement. These boundaries, visualized as distinct domains (Figure 3g), endow the material with a superior strength-ductility combination (160 MPa strength with 16% strain, Figure 3h) while also acting as efficient phonon scatterers. Consequently, the material achieves a maximum *ZT* of 0.6 at 400 K and a 45% enhancement in average *ZT* [60]. The twin boundary is a coherent interface where the crystal structure exhibits mirror symmetry [57]. Interestingly, nanoscale twins can induce contrasting mechanical effects in different materials. For example, in Mg_2_Si, nanoscale twins induce softening, reducing the theoretical shear strength from 6.88 to 0.93 GPa due to prestretched and weakened Mg–Si bonds at the interface [65]. Conversely, in Bi_2_Te_3_, first-principles calculations indicate that nanotwins enhance the ideal shear strength from 0.19 to 0.60 GPa. This strengthening originates from newly formed Te-Te covalent bonds near the twin boundaries, which reinforce the interlayer coupling within the quintuple layers [16]. Furthermore, nanotwin in InSb can reduce lattice thermal conductivity by 22.2%, confirming the potential of twin engineering for thermoelectric optimization [16].

### 3.3. Nanocomposites

Nanocomposites optimize both mechanical and thermoelectric properties through the strategic incorporation of nanoscale features such as nanoprecipitates [20], dual-phase heterostructures [22], and porous architectures [24]. These engineered nanocomposites not only enhance fracture toughness and damage tolerance through crack deflection, bridging, and blunting [25], but also serve as effective phonon scattering centers, significantly reducing lattice thermal conductivity [21]. For example, Mn alloying introduced high-density MnTe nanoprecipitates into the Sn_1.03−x_Mn_x_Te system (Figure 3i) [20]. The optimal Sn_0.93_Mn_0.10_Te composition achieves a 74.5% increase in yield strength (with precipitates contributing ~57.8 MPa) and enhanced fracture resistance through crack deflection and blunting (Figure 3j) [20]. Multi-component doping of SnTe with MgB_2_ and Sb generates a unique architecture comprising core–shell nanoparticles at grain boundaries and Sb-rich nanoprecipitates within grains. This architecture simultaneously increases mechanical strength and hardness by ~168–176% and suppresses lattice thermal conductivity to 0.35–0.61 W·m^−1^·K^−1^ through broad-spectrum phonon scattering [21]. The dual-phase heterostructure in the Cu_2−x_Se system enables simultaneous improvement of strength and ductility, as demonstrated in Figure 3l [22]. The soft *β*-phase acts as a strain carrier to enhance ductility, while the hard α-phase induces back stress and promotes geometrically necessary dislocations at phase boundaries (Figure 3k) [22]. The incorporation of porous architectures provides another effective route for modulating both thermal and mechanical properties in nanocomposites. For instance, a hierarchical porous structure with multi-scale pores and K_2_Ti_6_O_13_ nanowhiskers in Sn_1.03_Te creates a full-spectrum phonon scattering network, reducing the lattice thermal conductivity by 34.1% to 1.76 W·m^−1^·K^−1^ at room temperature [25,66].

Energy filtering is a key strategy for enhancing the power factor of thermoelectric materials. Its core principle involves the introduction of nanoscale interfaces, such as precipitates, phase boundaries, or heterojunctions, into the material to create a moderate potential barrier. This barrier selectively scatters low-energy carriers while allowing high-energy carriers to pass, thereby altering the energy distribution of carriers and enhancing the Seebeck coefficient without significantly compromising electrical conductivity. This leads to an optimized power factor [67]. The energy filtering effect has been confirmed in various material systems. In p-type BiSbTe, the introduction of Sb_2_O_3_ nanoparticles at grain boundaries creates energy-filtering barriers that not only enhance the power factor but, owing to the stable interface structure, also enable the material to retain its thermoelectric performance over 24 months of aging [68]. Introducing the wide-bandgap semiconductor ZnO into a SnTe matrix forms incoherent heterojunction interfaces. These interfaces not only act as energy filters for holes to enhance the Seebeck coefficient, but the lattice mismatch and defects also enhance phonon scattering, reducing the lattice thermal conductivity [69]. Similarly, adding Bi_2_O_3_ to SnTe generates numerous SnO_2_, Bi_2_O_3_, and Bi-rich nanoprecipitates, significantly improving the average power factor over a broad temperature range [70].

## 4. Flexible Thermoelectric Applications

### 4.1. Near-Room-Temperature Applications

Flexible thermoelectric devices, with advantages such as noiseless operation, high reliability, ease of miniaturization, and long service life, show great potential in power generation applications [71]. Depending on the application scenarios, existing flexible thermoelectric materials and devices can be classified into two categories: near-room-temperature and high-temperature applications. Among these, near-room-temperature flexible thermoelectric semiconductors are widely employed in power generation systems for small wearable devices and household appliances. These materials, which are typically fabricated in the form of nanowires or thin films, operate efficiently under small temperature gradients (Δ*T*).

**Figure 4:**
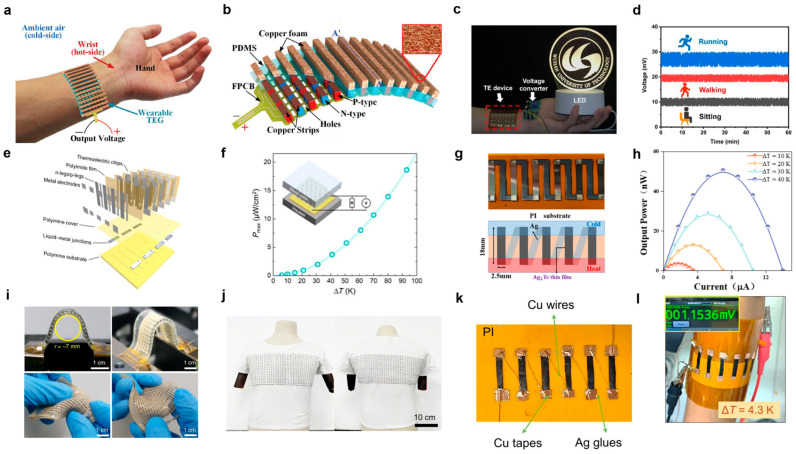
**Near-room-temperature applications of nanostructured semiconductors.** (**a**,**b**) The structural design of a wearable TEG is shown, with a copper foam heat sink integrated for use on the wrist [72] (Reprinted with permission from ref. [72]. Copyright 2018 IEEE). (**c**) An LED is illuminated by body heat under ambient conditions [47]. (**d**) The output voltage and operational stability of the Bi_2_Te_3_ thin-film thermoelectric device were evaluated under three common wearable scenarios: sitting, walking, and running [47] (Reprinted with permission from ref. [47]. Copyright 2025 Wiley-VCH). (**e**) The design and construction process of the TE device [78]. (**f**) The correlation between the maximum output power (*P_max_*) and the applied temperature gradient [78] (Reprinted with permission from ref. [78]. Copyright 2021 AAAS). (**g**) The bottom schematic and the upper optical photograph present the structure of the Ag_2_Te thermoelectric thin-film generator [81]. (**h**) The output power (*P_out_*) as a function of current under Δ*T* varied between 10 and 40 K [81] (Reprinted with permission from ref. [81]. Copyright 2024 MDPI). (**i**) The excellent conformability of the compliant TE devices under various deformation states [79] (Reprinted with permission from ref. [79]. Copyright 2020 Springer Nature). (**j**) Optical images showcasing a garment integrated with the thermoelectric textile [80] (Reprinted with permission from ref. [80]. Copyright 2023 Royal Society of Chemistry). (**k**,**l**) The six-legged device made from cold-pressed SWCNT films treated with NaBH_4_, inserted with the generated voltage while wearing the device [82] (Reprinted with permission from ref. [82]. Copyright 2024 Springer Nature).

Bi_2_Te_3_-based semiconductors are the most mature near-room-temperature thermoelectric materials. Researchers have developed a high-performance Bi_2_Te_3_-based flexible TE generator for body heat harvesting by incorporating copper foam as a heat sink (Figure 4a,b) [72]. At a 45 K temperature difference, the device achieves an output power of 276.3 μW with a superior power-to-weight ratio of 30.73 μW·g^−1^, outperforming traditional plate-fin designs [72]. Electrodeposited Bi_2_Te_3_ and Te nanowire networks in commercial polyester templates enable flexible thermoelectric devices [73]. A novel Bi_2_Te_3_ material with the ZnO nanowire structure achieves a record 6.57% conversion efficiency in thermoelectric modules. This design delivers high power density at small temperature differences while reducing material consumption by 90%, offering a cost-effective solution for flexible thermoelectric applications [74]. Notably, a flexible Bi_2_Te_3_ thin-film TE device comprising 44 p-n legs delivers a maximum power of 1.2 μW (Δ*T* = 120 K) and maintains a stable output voltage of 210 mV for over 720 s at Δ*T* = 200 K (Figure 4c,d) [47]. Furthermore, the device withstands 10,000 bending cycles with minimal resistance variation (<5%), demonstrating exceptional mechanical durability and promising potential for wearable energy harvesting applications [47].

Three-dimensional (3D) Bi_2_Te_3_-based flexible thermoelectric devices can be fabricated through rational architectural design. For instance, a 3D-printed generator achieves 127.9 mV and 0.49 μW at Δ*T* = 40 K, functioning as a self-powered sensor for smart homes [75]. Cold-press sintering provides an alternative fabrication route, producing devices that exhibit both a high *ZT* and low thermal conductivity [76]. Optimization extends to leg geometry, where a general design principle achieved a 466% efficiency gain using 67% less material, enabling a high-performance wearable TE generator with a soft-rigid structure [77]. Further innovations include a reconfigurable, self-healing, and recyclable wearable TE generator (Figure 4e,f) [78], and a compliant generator that integrates soft conductors and stretchable electrodes to deliver 7.02 mW while conforming to 3D surfaces (Figure 4i) [79]. A durable and recyclable thermoelectric textile demonstrates real-world value by generating sufficient power for a cell phone and providing personal cooling, highlighting its dual function in wearable energy harvesting and thermal management (Figure 4j) [80].

In addition to Bi_2_Te_3_-based semiconductors, several other TE material systems show significant promise for flexible thermoelectrics. Ag_2_Te-based materials, for instance, include surfactant-free nanowires with a low thermal conductivity of 0.22 W·m^−1^·K^−1^. An optimized annealing process is proposed for co-sputtered Ag_2_Te films, which further enables a high power factor of ~7.85 μW·cm^−1^·K^−2^ by tuning stoichiometry and refining the microstructure (Figure 4g,h), while also enhancing mechanical adhesion [81]. Turning to carbon-based materials, a triple-optimized single-walled carbon nanotube (SWCNT) film achieves a high power factor of 20.29 µW·cm^−1^·K^−2^ (Figure 4k,l). A corresponding six-leg device delivers a power density of 2996 µW·cm^−2^ at Δ*T* = 40 K and retains a stable resistance (<5% change) after 2000 bending cycles [82]. Furthermore, a hot-pressed Te/PEDOT: PSS composite film reaches a power factor of 149 μW·m^−1^·K^−2^ with excellent flexibility. It has been successfully used in a robotic temperature sensor capable of distinguishing between hot and cold objects, underscoring its potential for intelligent electronics [83].

### 4.2. High-Temperature Applications

**Figure 5:**
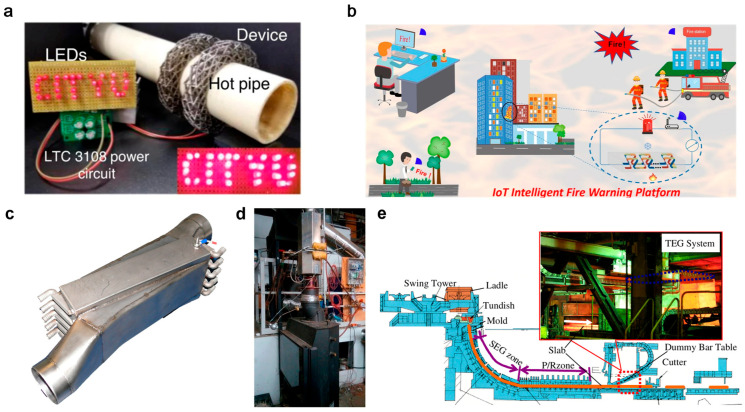
**High-temperature applications of nanostructured semiconductors.** (**a**) A magnified optical micrograph of the developed planar-architected thermoelectric generator (TEG) [85] (Reprinted with permission from ref. [85]. Copyright 2023 Springer Nature). (**b**) The high-performance SWCNT-based thermoelectric composite enables an intelligent fire warning platform for urban buildings [86] (Reprinted with permission from ref. [86]. Copyright 2024 Springer). (**c**) A prototype of a TEG designed for passenger cars [88] (Reprinted with permission from ref. [88]. Copyright 2016 Springer). (**d**) The TEG is installed on the flue gas channel of the stove [89]. (**e**) A thermoelectric generation system integrated into the continuous casting line [89] (Reprinted with permission from ref. [89]. Copyright 2014 Springer).

High-temperature thermoelectric semiconductors exhibit extensive applications in industrial waste heat recovery and automotive exhaust heat utilization, operating effectively under large temperature gradients inherent to these application scenarios. For example, using a 3D printing process incorporating 4% organic binder, SnSe bulk material achieves a record *ZT* of 1.7 for printed thermoelectrics, enabling the first fully printed medium-to-high temperature SnSe devices and establishing a low-cost, scalable route for high-performance thermoelectric conversion [84]. Three-dimensional core–shell microlattice thermoelectric generators fabricated via digital light processing and partial carbonization demonstrate ~10% power conversion efficiency and ~1.4 μW maximum power output under a 120 K temperature difference. These devices exhibit exceptional compressive ductility (>50% strain) with specific energy absorption of ~30 J·g^−1^, conforming effectively to curved heat sources while powering electronic circuits, showing groundbreaking potential for sustainable waste heat recovery (Figure 5a) [85]. Flexible SWCNT/TiC composite films prepared through solution-mixing and vacuum filtration enabled thermoelectric devices with an ultrafast fire warning response of ~0.1 s, adjustable threshold (1–10 mV), excellent repeatability over 50 cycles, and long-term durability after 180 days (Figure 5b) [86]. First-principles calculations reveal that polyyne chains maintain exceptional thermomechanical stability at 900 K while retaining *ZT* > 1 under strain [87]. This combination of high thermoelectric performance and mechanical robustness positions this one-dimensional carbon material as a promising candidate for stable integration in next-generation flexible and micro-nano thermoelectric systems. In industrial applications, systems directly converting waste heat to grid electricity show substantial potential for energy savings and CO_2_ emission reduction. BiTe thermoelectric generators integrated into vehicle exhaust systems delivered 150 W in passenger cars and 416 W in heavy-duty trucks (Figure 5c) [88]. A Bi-Te thermoelectric generation system developed for radiant waste heat recovery from continuous casting slabs (~1188 K) in steelworks (Figure 5d,e) achieved approximately 9 kW output with power density roughly ten times that of solar power systems [89]. These compact systems show considerable potential for automotive fuel savings and carbon reduction, though module temperature tolerance and efficiency require further improvement.

Oxidation poses a critical challenge to the long-term reliability and operational lifetime of flexible thermoelectric devices in practical applications. Indeed, oxidation resistance is a fundamental requirement alongside mechanical durability in device design. To address this issue, extensive studies have been conducted to enhance material stability through both material innovation and process optimization. In the inorganic Bi_2_Te_3_ system, Sb_2_Te_3_-based nanosolder combined with N_2_ sintering can form a protective thiol passivation layer, resulting in only a 3.6% change in electrical resistivity after six months of air exposure [90]. In contrast, unmodified Sb_2_Te_3_ films begin oxidizing above 100 °C and fully convert to detrimental oxide phases (Sb_2_O_4_ and TeO_2_) after annealing at 250 °C for 15 h, leading to severe degradation of electrical conductivity and power factor [91]. Interestingly, the oxidation process of Sb_2_Te_3_ on single-walled carbon nanotube (SWCNT) substrates can serve as an effective n-type doping strategy, achieving a high power factor of up to 2440 μW·m^−1^·K^−2^ alongside excellent mechanical flexibility (resistance change <2% after 500 bending cycles) [92].

Annealing conditions play a critical role in modulating the electronic properties of flexible thermoelectric devices by tailoring microstructure, phase composition, and defect states. For Ti-doped ZnO films, vacuum annealing at 300 °C optimally relieves compressive stress, enhancing carrier mobility and density-of-states effective mass to achieve a peak power factor of 19.10 μW·m^−1^·K^−2^ [93]. Similarly, annealing Ag_2_Te films at 200 °C optimizes the Ag/Te ratio toward stoichiometry, significantly boosting carrier mobility to ~3291 cm^2^ V^−1^ s^−1^ and increasing the room-temperature power factor by 21%. Furthermore, mechanical configuration during annealing can mitigate substrate-related constraints. Annealing Bi_2_Te_3_ films under a concave bent condition counteracts polyimide substrate shrinkage, promoting larger crystallites and preferred orientation, which enhances electrical conductivity and yields a power factor of 16.1 μW cm^−1^ K^2^ [94]. Therefore, the optimization of annealing parameters, including atmosphere, temperature, duration, and mechanical configuration, is essential for tailoring electronic transport and maximizing thermoelectric efficiency in flexible devices.

## 5. Conclusions and Outlook

This review systematically elucidates the role of nanostructural design in optimizing both the thermoelectric and mechanical properties of semiconductors. On one hand, low-dimensional nanostructures can induce a brittle-to-ductile transition while concurrently optimizing the transport of charge carriers and phonons. On the other hand, microstructural control through defect engineering, including dislocations, boundaries, and nanocomposites, significantly strengthens and toughens while preserving or even enhancing their thermoelectric performance. These strategies have been successfully implemented in flexible thermoelectric applications, including wearable devices and waste heat recovery systems. Looking forward, several key challenges require further investigation. First, a deeper understanding of the dynamic evolution of material microstructures under multi-field coupling conditions and their correlation with macroscopic properties is essential. Second, developing advanced manufacturing processes for the scalable production of materials that combine high thermoelectric figure of merit with mechanical reliability remains crucial. Furthermore, interface optimization at the device level, thermomechanical stability, and long-term service reliability represent core issues for industrial implementation. Through the integration of cross-scale material design, advanced fabrication technologies, and intelligent device integration, high-performance flexible thermoelectric technology is poised to open new application prospects in addressing global energy sustainability challenges.

## Figures and Tables

**Table 1:** Comparison of mechanical properties, *ZT*, and power factor (PF) of different materials in the form of nanowires, pillars, and thin films under corresponding loading modes and temperatures (*T*) [6,9,31,33,35,36,38,41,42,43,44,45,47,49].

Materials	Configuration	Dimension	Loading Mode	Mechanical Properties	*T* (K)	PF (μW cm^−1^ K^−2^)	*ZT*
Ag_2_Te [6]	Nanowire	330 nm (Diameter) × 740 nm (Length)	Tension	75.7% (Fracture Strain)	/	/	/
Ag_2_Te [33]	Nanowire	50–100 nm (Diameter)	Tension	~11.3 MPa (Tensile Strength)	300	3.62	0.25
Si [36]	Nanowire	20 nm (Diameter) × 20 nm (Length)	/	/	200	/	1.0
SiGe [35]	Nanowire	64 ± 11 nm (Diameter)	/	/	/	/	/
SnSe [38]	Pillar	600 nm (Diameter)	Compression	1.15 GPa (Yield Strengh) 7.3% (Strain)	/	/	/
Si [31]	Pillar	1000 nm (Diameter)	Compression	10.8 GPa (Engineering Stress) 10% (Strain)	/	/	/
Bi_2_Te_3_ [47]	Thin films	1500 nm (Thickness)	Bending	10,000 cycles (r = 4 mm)	550	27.6	/
Bi_2_Te_3_ [9]	Thin films	12,000 mm (Thickness)	Bending	>1000 cycles (r = 5 mm)	300	46	0.9
Ag_2_Se [49]	Thin films	15,000 mm (Thickness)	Bending	>1000 cycles (r = 5 mm)	303	25.7	1.06
Ag-Bi_2_Te_3_ [41]	Thin films	468.9 nm (Thickness)	Bending	2000 cycles (r = 18 mm)	300	20.6	1.2
Ag-Bi_0.5_Sb_1.5_Te_3_ [42]	Thin films	750 nm (Thickness)	Bending	1000 cycles (r = 5 mm)	420	14.0	/
Ag_2_Se [43]	Thin films	200 nm (Thickness)	Bending	500 cycles (r = 5 mm)	300	26.3	1.0
Cu_2_Se [44]	Thin films	500 nm (Thickness)	Bending	1000 cycles (r = 5 mm)	548	19.2	0.76
Ce-Fe_3_CoSb_12_ [45]	Thin films	300 nm (Thickness)	Bending	2000 cycles (r = 5 mm)	653	10	0.6

## Data Availability

The original contributions presented in this study are included in the article. Further inquiries can be directed to the corresponding authors.

## Abbreviations

The following abbreviations are used in this manuscript:

TE	Thermoelectric
TEM	Transmission electron microscopy
SEM	Scanning electron microscopy
STEM	Scanning transmission electron microscopy
2D	Two-dimensional
3D	Three-dimensional
TEG	Thermoelectric generator
SWCNT	Single-walled carbon nanotube
